# Supplementary material for: Incidence of lab-confirmed dengue fever in a pediatric cohort in Delhi, India
Source: PLoS Negl Trop Dis. 2022 Apr 7;16(4):e0010333. doi: 10.1371/journal.pntd.0010333 (PMC9017938; doi:10.1371/journal.pntd.0010333)
Supplement: S1 Appendix — Table A: Distribution of different dengue assay results and its classification. Table B: Clinical characteristics and outcomes of acute primary, acute secondary and past dengue infection. (DOCX) [file pntd.0010333.s001.docx]

**Incidence of lab-confirmed dengue fever in a pediatric cohort in Delhi, India**

Bireshwar Sinha, Nidhi Goyal, Mohan Kumar, Aashish Choudhary, Alok Arya, Anitha Revi, Ankita Dutta, Deepak More and Temsunaro Rongsen-Chandola

**Online Supplementary Material**

**Contents**

Table A: Distribution of different dengue assay results and its classification

Table B: Clinical characteristics and outcomes of acute primary, acute secondary and past dengue infection.

**Table A: Distribution of different dengue assay results and its classification***

| **Dengue assays** | **Scenario 1** | **Scenario 2** | **Scenario 3** | **Scenario 4** | **Scenario 5** | **Scenario 6** |
| --- | --- | --- | --- | --- | --- | --- |
| NS1 | + | - | + | - | + | - |
| IgM | + | + | - | + | + | - |
| IgG | + | + | - | - | - | + |
| Number of cases | 3 | 3 | 45 | 23 | 0 | 29 |
| Total | 6 | | 68 | | | 29 |
| Category | Acute dengue fever - Secondary | | Acute dengue fever - Primary | | | Past infection |

*The median (IQR) time of blood collection was on day 3 (3 to 4) from onset of fever

**Table B: Clinical characteristics and outcomes of acute primary, acute secondary and past dengue infection.**

| **Description** | **Acute dengue infection (N = 74)**  **n (%)** | **Past infections (N = 29)**  **n (%)** |
| --- | --- | --- |
| **Clinical symptoms (other than fever) ^a^**  Cough/cold  Abdominal pain  Vomiting  Others  Decreased appetite  Joint pain | 3 (0.04)  1 (0.01)  1 (0.01)  1 (0.01)  1 (0.01)  1 (0.01) | 1 (0.03)  2 (0.07)  1 (0.03)  1 (0.03)  1 (0.03)  1 (0.03) |
| Duration of episode in days: Mean (SD) | 6.87 (1.65) | 7.72 (2.55) |
| Highest temperature in Fahrenheit during an episode: Mean (SD) | 103.0 (0.00) | 101.1 (2.48) |
| **Outcome of Dengue fever episodes**  Recovered  Recovered with Complications ^b^  Death | 74 (100)  0 (0)  0 (0) | 29 (100)  0 (0)  0 (0) |
| ^a^ There were no reports of breathlessness, diarrhea, constipation, blood in stool, jaundice, headache, seizure, altered sensorium, rash, sore throat, and malaise.  ^b^ There were no reports of any complications including GI bleeding, intestinal perforation, encephalopathy, myocarditis, hemodynamic shock, hepatitis, and renal impairment. | | |
