# Supplementary material for: Incidence of lab-confirmed dengue fever in a pediatric cohort in Delhi, India
Source: PLoS Negl Trop Dis. 2022 Apr 7;16(4):e0010333. doi: 10.1371/journal.pntd.0010333 (PMC9017938; doi:10.1371/journal.pntd.0010333)
Supplement: Attachment — Submitted filename: PNTD_D_21_01656_R1_reviewer.pdf [file pntd.0010333.s005.pdf]

PNTD-D-21-01656_R1_reviewer.pdf

WORD COUNT 10541
TIME SUBMITTED 03-MAR-2022 08:04AM
PAPER ID 83230609

PLOS Neglected Tropical Diseases

Incidence of lab-confirmed Dengue fever in a pediatric cohort in North India

--Manuscript Draft--

**Manuscript Number:**	PNTD-D-21-01656R1
**Full Title:**	Incidence of lab-confirmed Dengue fever in a pediatric cohort in North India
**Short Title:**	Incidence of Dengue fever in North Indian children
**Article Type:**	Research Article
**Keywords:**	Dengue fever; incidence; epidemiology; children; cohort; North India
**Abstract:**	Background: Our aim was to estimate the overall and age-specific incidence of lab-confirmed dengue fever using ELISA based assays among children 6 months to 15 years in Delhi.<br>Methods: We enrolled a cohort of 984 children aged 6 months to <14 years in South Delhi and followed-up weekly for fever for 24 months or till 15 completed years of child-age. Households of the enrolled children were geo-tagged. NS1, IgM and IgG assays were conducted using ELISA method to confirm dengue fever in children with ≥3 consecutive days of fever. Molecular typing was done in a subset of NS1 positive cases to identify the circulating serotypes.<br>Principal Findings: We had a total of 1953 person-years (PY) of follow up. Overall, there were 4208 episodes of fever with peaks during June to November. The overall incidence (95%CI) of fever was 215/100 PY (209 to 222). A total of 74/1250 3-day fever episodes were positive for acute dengue fever (NS1 and/or IgM positive). The overall incidence (95%CI) of acute dengue fever was 37.9 (29.8 to 47.6) per 1000 PY; highest among children aged 5 to 10 years (50.4 per 1000 PY, 95% CI 36.5 to 67.8). Spatial autocorrelation analysis suggested a clustering pattern for the dengue fever cases (Moran's Index 0.35, z-score 1.8, p=0.06). Dengue PCR was positive in 16 of the 24 specimens tested; DEN 3 was the predominant serotype identified in 15/24 specimens.<br>Conclusions: We found a high incidence of dengue fever among under 15-year children with clustering of cases in the community. DEN 3 was the most commonly circulating strain encountered. The findings underscore the need for development of affordable pre-vaccination screening strategy as well as newer dengue vaccines for young children while continuing efforts in vector control.
**Additional Information:**	
**Question**	**Response**
**Financial Disclosure**<br><br>Enter a financial disclosure statement that describes the sources of funding for the work included in this submission. Review the submission guidelines for detailed requirements. View published research articles from *PLOS NTDs* for specific examples.<br><br>This statement is required for submission and **will appear in the published article** if the submission is accepted. Please make sure it is accurate.	The authors have received no funding for this specific work presented.

Powered by Editorial Manager® and ProduXion Manager® from Aries Systems Corporation

**Unfunded studies**<br><br>Enter: *The author(s) received no specific funding for this work*.<br><br>**Funded studies**<br><br>Enter a statement with the following details:<br>• Initials of the authors who received each award<br>• Grant numbers awarded to each author<br>• The full name of each funder<br>• URL of each funder website<br>• Did the sponsors or funders play any role in the study design, data collection and analysis, decision to publish, or preparation of the manuscript?<br>• **NO** - Include this sentence at the end of your statement: *The funders had no role in study design, data collection and analysis, decision to publish, or preparation of the manuscript*.<br>• **YES** - Specify the role(s) played.<br><br>* typeset	
**Competing Interests**<br><br>Use the instructions below to enter a competing interest statement for this submission. On behalf of all authors, disclose any competing interests that could be perceived to bias this work—acknowledging all financial support and any other relevant financial or non-financial competing interests.<br><br>This statement **will appear in the published article** if the submission is accepted. Please make sure it is accurate. View published research articles from *PLOS NTDs* for specific examples.	The authors have declared that no competing interests exist.

Powered by Editorial Manager® and ProduXion Manager® from Aries Systems Corporation

**NO authors have competing interests**<br><br>Enter: *The authors have declared that no competing interests exist.*<br><br>**Authors with competing interests**<br><br>Enter competing interest details beginning with this statement:<br><br>*I have read the journal's policy and the authors of this manuscript have the following competing interests: [insert competing interests here]*<br><br>* typeset<br><br>This statement is required for submission and **will appear in the published article** if the submission is accepted. Please make sure it is accurate and that any funding sources listed in your Funding Information later in the submission form are also declared in your Financial Disclosure statement.	
**Data Availability**<br><br>Authors are required to make all data underlying the findings described fully available, without restriction, and from the time of publication. PLOS allows rare exceptions to address legal and ethical concerns. See the PLOS Data Policy and FAQ for detailed information.	No - some restrictions will apply

Powered by Editorial Manager® and ProduXion Manager® from Aries Systems Corporation

A Data Availability Statement describing where the data can be found is required at submission. Your answers to this question constitute the Data Availability Statement and **will be published in the article**, if accepted.<br><br>**Important:** Stating 'data available on request from the author' is not sufficient. If your data are only available upon request, select 'No' for the first question and explain your exceptional situation in the text box.<br><br>Do the authors confirm that all data underlying the findings described in their manuscript are fully available without restriction?	
**Describe where the data may be found in full sentences. If you are copying our sample text, replace any instances of XXX with the appropriate details.**<br><br>• If the data are **held or will be held in a public repository**, include URLs, accession numbers or DOIs. If this information will only be available after acceptance, indicate this by ticking the box below. For example: *All XXX files are available from the XXX database (accession number(s) XXX, XXX.).*<br>• If the data are all contained **within the manuscript and/or Supporting Information files**, enter the following: *All relevant data are within the manuscript and its Supporting Information files.*<br>• If neither of these applies but you are able to provide **details of access elsewhere**, with or without limitations, please do so. For example:<br><br>*Data cannot be shared publicly because*	Data will be made available for the primary purpose to substantiate the presented findings.

Powered by Editorial Manager® and ProduXion Manager® from Aries Systems Corporation

of [XXX]. Data are available from the XXX Institutional Data Access / Ethics Committee (contact via XXX) for researchers who meet the criteria for access to confidential data.<br><br>*The data underlying the results presented in the study are available from (include the name of the third party and contact information or URL).*<br>• This text is appropriate if the data are owned by a third party and authors do not have permission to share the data.<br><br>* typeset	
Additional data availability information:	Tick here if your circumstances are not covered by the questions above and you need the journal's help to make your data available.

Powered by Editorial Manager® and ProduXion Manager® from Aries Systems Corporation

Incidence of lab-confirmed dengue fever in a pediatric cohort in Delhi, India

Authors and Affiliations

Bireshwar Sinha[1,2], Nidhi Goyal[1], Mohan Kumar[1], Aashish Choudhary[3], Alok Arya[1], Anitha Revi[1], Ankita Dutta[4], Deepak More[4] and Temsunaro Rongsen-Chandola[1*]

[1]Centre for Health Research and Development, Society for Applied Studies, New Delhi-110016

[2]Clinical and public health fellow, DBT/Wellcome India Alliance, Hyderabad-5000034

[3]Virology Lab, Department of Microbiology, All India Institute of Medical Sciences, New Delhi-110026

[4]Clinical and Research Laboratories, Society for Applied Studies, New Delhi-110016

***Corresponding Author**

Temsunaro Rongsen Chandola

Senior Scientist and Deputy Director

45, Kalu Sarai, Centre for Health Research and Development Society for Applied Studies, New Delhi-110016

Email: naro@sas.org.in

Running Title: Incidence of dengue fever in children from Delhi, India

Type of Article: Original

Abstract

Background: Our aim was to estimate the overall and age-specific incidence of lab-confirmed dengue fever using ELISA based assays among children 6 months to 15 years in Delhi.

Methods: We enrolled a cohort of 984 children aged 6 months to <14 years in South Delhi and followed-up weekly for fever for 24 months or till 15 completed years of child-age. Households of the enrolled children were geo-tagged. NS1, IgM and IgG assays were conducted using ELISA method to confirm dengue fever in children with ≥3 consecutive days of fever. Molecular typing was done in a subset of NS1 positive cases to identify the circulating serotypes.

Principal Findings: We had a total of 1953 person-years (PY) of follow up. Overall, there were 4208 episodes of fever with peaks during June to November. The overall incidence (95%CI) of fever was 215/100 PY (209 to 222). A total of 74/1250 3-day fever episodes were positive for acute dengue fever (NS1 and/or IgM positive). The overall incidence (95%CI) of acute dengue fever was 37.9 (29.8 to 47.6) per 1000 PY; highest among children aged 5 to 10 years (50.4 per 1000 PY, 95% CI 36.5 to 67.8). Spatial autocorrelation analysis suggested a clustering pattern for the dengue fever cases (Moran's Index 0.35, z-score 1.8, p=0.06). Dengue PCR was positive in 16 of the 24 specimens tested; DEN 3 was the predominant serotype identified in 15/24 specimens.

Conclusions: We found a high incidence of dengue fever among under 15-year children with clustering of cases in the community. DEN 3 was the most commonly circulating strain encountered. The findings underscore the need for development of affordable pre-vaccination screening strategy as well as newer dengue vaccines for young children while continuing efforts in vector control.

Key Words: Dengue fever, incidence, Children, Cohort, India

Author summary

What is already known

South Asian countries especially the Indian subcontinent contributes the highest to the global burden of dengue.

The number of dengue cases reported in India is likely an underestimate of the actual disease burden and there is a clear lack in the availability of population-based data on incidence of dengue in India.

What this study adds

In our pediatric cohort of 984 children aged 6 months to 15 years from Delhi, India, we found a high incidence of lab-confirmed dengue fever, with the highest burden among 5 to 10 year old children. Dengue fever was observed to be highest in the post-monsoon months with significant clustering of cases in the community. DEN 3 was the most commonly circulating strain encountered.

Given the high burden in children, the findings highlight the need for strengthening efforts to developing newer dengue vaccines for younger children.

Introduction

The World Health Organization listed dengue as one of the top 10 potential threats to global health in 2019. Global burden of dengue was estimated to be 104 million (95% CI 64 to 159) cases in 2017(1) with the highest burden in South Asia of 3546.9 cases (95% Uncertainty interval 2128.5 to 5429.5) per 100,000 population.(1) The number of dengue related deaths, globally, is estimated to increase from 6957 (95% UI 7613 to 30091) in 1990 to 40467 (95% UI 17620 to 49778) in 2017.(1) Clinically apparent dengue can affect all age groups but is most frequently observed in the age group 5-15 years, globally and the highest mortality is in under-five children.(2) Modeling of surveillance data suggest, India contributes to around one-third of the global burden of symptomatic dengue infections (3).

In India, during last two decades, large and frequent dengue outbreaks and urban to rural spread with increasing proportion of severe cases, along with hyper endemicity in urban areas are reported (4). In 2017, National Vector Borne Disease Control Program (NVBDCP) reported 188,401 laboratory confirmed cases of dengue and 325 deaths through its network of 646 sentinel surveillance hospitals (5, 6). It is speculated that the number of dengue cases is highly underreported in India (7). A recent systematic review in 2018 estimated that the proportion of laboratory confirmed dengue cases on testing of 213,285 clinically suspected patients from 180 studies in India was 38.3% (95% CI 34.8% to 41.8%).(8) The overall seroprevalence of dengue fever in India based on 7 studies was 56.9% (95% CI 37.5% to 74.4%). The systematic review observed a clear lack in the availability of population-based data on incidence of dengue in India and highlighted the need of community-based studies for burden estimation (8).

Dengue prevention and control has been largely based on principles of vector control. However, currently several new dengue vaccines are in development. In December 2015, the first dengue

vaccine (CYD-TDV, or Dengvaxia®) was registered and is now licensed in several countries including Mexico and Philippines. The WHO in its 2016 position paper on vaccination against dengue recommends its use only in children 9 years and above in high burden countries. With newer indigenous vaccines being developed, it is imperative to have population-based incidence data on dengue for conducting vaccine studies and designing vaccination strategies. Our study was planned with the primary objective to estimate the incidence of lab-confirmed acute dengue fever using ELISA based assays in children under 15 years of age. In addition, we planned to report the age-categorized incidence in children aged <5 years, 5-10 years, and 10-15 years of age. As an exploratory objective we reported the most common circulating serotype in the population in a subsample.

Methods

Research Ethics Approval

The cohort study was approved by the Ethics Review Committee, Centre for Health Research and Development Society for Applied Studies, New Delhi, India in 2017.

Study population

The study was conducted as a sub-cohort within the Surveillance of Enteric Fever in India (SEFI) cohort among children in Delhi (CTRI/2017/09/009719)(9), in two contiguous blocks of Sangam Vihar, a low-middle income urban neighborhood in the southern district.

A door-to-door survey was conducted in the two blocks to line list the households with potentially eligible children. Families were screened to determine likelihood of moving out of the study area and/or adhering to study procedures specially blood specimen collection. Eligible children aged between 6 months and <14 years who were likely to stay in the study area for two

years were enrolled consecutively in the cohort from contiguous households until the sample size was attained. Prior to screening, written informed consent for participation and storage of specimens for future research was obtained from the parents after explaining study processes. We obtained verbal assent from children aged 7 to 12 years and written assent from children aged above 12 years. All enrolled children were followed up for 24 months or until 15 years of age, whichever was earlier (Fig 1).

Figure 1. Study Flowchart

Acute febrile illness surveillance

We conducted active weekly contacts either through phone or home visit with at least one face-to-face contact every four weeks to collect information on fever, illness or hospitalizations. A monthly mobile recharge of INR 50 was provided to all enrolled families to encourage early telephonic reporting of fever to the study team. Families were provided with a digital thermometer and fever diary card to document temperature. Fever was defined as caregiver reported or a recorded temperature of $\geq 38^0$C (100.4^0 F). Upon reporting of fever, our study team visited the participant's home as early as possible. Participant was visited daily during the fever episode. If fever lasted for ≥3 days, the child was referred to the study fever clinic at Hakeem Abdul Hameed Centenary Hospital (HAHCH) for physician evaluation and blood specimen collection (not later than 6 days of fever onset) to test for enteric fever (blood culture) and dengue fever. Management of the fever episode was as per the advice of the physician in the study fever clinic or any other preferred place of care-seeking. In case the participant visited other health facilities for care-seeking, information related to the fever episode was collected during the weekly contacts.

Laboratory methods

Blood specimens were transported to Clinical and Research Laboratories, Society for Applied Studies (CRL SAS), New Delhi, within 2 hours of collection. Serum was separated in CRL SAS and used for the laboratory assays for dengue using the automated EVOLIS™ Twin Plus system (BioRad, California, USA). Dengue NS1 Ag Microlisa, dengue IgM Microlisa and dengue IgG Microlisa (J. Mitra & Co. Pvt. Ltd., New Delhi, India) kits were used.

To study the circulating serotypes of dengue viruses, the serum specimens positive for NS1 antigen with an optical density (OD) value of ≥1.8 were further used for molecular typing. This was based on prior experience that it is difficult to get a positive signal for a specific dengue serotype in multiplex PCR assays in specimens with lower NS1 OD values. The molecular typing of dengue viruses was conducted at the Virology lab of All India Institute of Medical Sciences, New Delhi using the Geno-Sen's® dengue 1-4 (Rotor Gene) real time PCR quantitative kit (Genome Diagnostics Pvt. Ltd.).

Data management

Data was collected on tablets using android application package with built-in Geographic Information Systems (GIS) developed in house by the SEFI team and stored in a secured Amazon cloud-based server. Data was monitored using a dashboard-based system; weekly reports were generated and reviewed. A comprehensive audit trail was maintained. Quality assurance was done by an independent team of experts.

Statistical analysis

To capture an incidence of acute dengue fever as low as 3 per 100 person-years (PY) of follow up among children under 15 years of age with 95% confidence, 30% relative precision, and 25% loss to follow up a total of 1841 PY of follow up was deemed to be sufficient. Based on our

previous experience and considering each child will contribute to around 1.9 years of follow up, a total of 969 eligible children were deemed to be required.

Analysis was done using STATA 16.0 MP (TX, USA). The Stata command “xtset” was used to declare the dataset to be panel data. The characteristics of the study population was presented as proportions (%) and mean (SD). ‘Person-year’ for each child was calculated from date of enrolment to censorship (end of study, withdrawal, death, or no face-to-face contact for ≥90 days). During the follow-up, if a participant was not contactable for ≥2 consecutive weeks, the information from the previous 2-weeks from the date of subsequent contact was captured and considered in person-years of follow up and the intervening time period was not considered in analysis.

Fever episodes positive for dengue NS1 and/or IgM were defined as lab-confirmed acute dengue fever episodes. In addition to NS1 and/or IgM, fever episodes that were positive for dengue IgG were identified as acute secondary dengue fever and those negative for IgG were acute primary dengue fever. Children were divided into 3 age categories i.e., 6 months to <5 years, 5 years to <10 years and 10 to 15 years. The age was calculated from date of birth verified from birth certificate or immunization card. The age-specific incidence rates of acute dengue fever or acute febrile illness was calculated as the number of new events in the specific age-interval divided by total number of person-years of follow-up contributed by all children at risk in this interval with allowing for children to move to higher age categories. Confidence intervals were calculated using the “poisson” option in Stata. The cases of lab-confirmed dengue fever were plotted using ArcGIS 10.8 on a base map using Sentinel 2 satellite data with 10 m spatial resolution available from Copernicus Open Access Hub (https://scihub.copernicus.eu/) of 2021 for the study area. After preparing the GIS maps, Moran’s Index of spatial autocorrelation was estimated to identify any clustering pattern of the dengue fever cases.

Results

We followed up a fixed cohort of 984 children from 755 households from the date of first enrollment, 24th October 2017 to date of completed follow up, 13th February 2020. In this cohort we had a total of 1953 person-years (PY) of follow up, of which, 535, 854 and 564 PY were contributed by children aged 6 months to <5 years, 5 to <10 and 10 to 15 years, respectively (Figure 1). During this follow-up period a total of 16 episodes of hospitalizations were reported in this pediatric cohort.

Characteristics of the study population

Around two-third (72%) of the children enrolled belonged to nuclear families with median monthly income of 143 USD; 77% of these households were overcrowded (Table 1). Piped water from the Delhi municipal corporation (81%) and bottled water (12%) were the major sources of household drinking water. Almost all used sanitary toilets, of which, one-third has flush systems; 16% of the households shared toilet with other families (Table 1).

Table 1. Characteristics of the study population

**Baseline characteristics of enrolled children**	**n/N (%) [a]**
Sex of the Child: Female	474/984 (48.17)
Age at enrollment	
6 months to < 5years	342/984 (34.76)
5 years to <10 years	434/984 (44.11)
10 years to <14 years	208/984 (21.13)
**Sociodemographic parameters**	
Type of Family	
Nuclear	540/755 (71.52)
Three generation	47/755 (6.23)
Joint	168/755 (22.25)
Family Size: Mean (SD)	5.83 (2.11)
Highest education in family as years of schooling: Mean (SD)	11.05 (3.17)
Type of House [b]	
Pucca	749/755 (99.21)
Mixed	6/755 (0.79)
Kuccha	0/755 (0.00)
Overcrowding present (>2.5 persons per living room)	583/755 (77.22)

Separate kitchen available		535/755 (70.86)
Primary fuel used for cooking in household		
	LPG Gas	754/755 (99.87)
	Others	1/755 (0.13)
Monthly income in USD [c]		
Median (IQR)		142.86 (114.29, 214.29)
Mean (SD)		192.76 (159.64)
**WASH parameters**		
Source of Drinking water		
	Piped water in household	609/755 (80.66)
	Bottled water	88/755 (11.66)
	Public tap/standpipe	34/755 (4.50)
	Tube well	7/755 (0.93)
	Tanker-truck	16/755 (2.12)
	Others	1/755 (0.13)
Practice of water treatment before drinking [d]		298/755 (39.47)
Type of Toilet facility used		
	Flush toilet	236/755 (31.26)
	Pit latrine	509/755 (67.42)
	Ventilated improved pit latrine	10/755 (1.32)
Sharing of toilet with other households		119/755 (15.76)

[a] This is a cohort of 984 children from 755 households – the denominators are mentioned accordingly
[b] A pucca house is one, which has walls and roof made of bricks, stones packed with cement. Kutcha houses are made of material other than those mentioned above, such as bamboos, mud, grass, reeds, thatch, etc.
[c] 1 US dollar (USD) = 70 Indian Rupee (INR)
[d] Methods of water treatment include boiling, chlorination, and filtration

Acute febrile illness surveillance

In this cohort, there were 4208 episodes of fever with peaks during the months of June to November (Table 2, Fig 2). The overall incidence (95%CI) of fever was 215/100 PY (209 to 222), with highest incidence in under-five children 607.6/100 PY (587.5 to 628.2). Median (IQR) duration of a fever episode was 2 (1-4) days. In 34.7% (1460/4208) of the fever episodes an antibiotic was prescribed which was initiated around day 2 (IQR 1 to 3) of fever onset. Oral beta-lactam, cephalosporin and macrolides were the most prescribed antibiotics. Of the 1504 episodes of 3-day fevers, dengue tests were conducted in 1250 episodes (83.1%, Figure 1). The median (IQR) time of blood collection was on day 3 (3 to 4) from onset of fever. In the other 3-day fever episodes (254/1504) dengue tests could not be performed due to refusal by parent for blood collection or family unavailable in the study area.

Table 2. Incidence of fevers by age-categories in the cohort (n = 984)

Age group	Person years of follow up	Number of Fever episodes	Incidence (95%CI) of fever per 100 PY	Number of ≥3-day fever episodes	Incidence (95% CI) of ≥3-day fevers per 100 PY
6m to <5y	535.08	3251	607.6 (587.5 to 628.2)	572	106.9 (98.4 to 115.9)
5y to <10y	853.84	547	64.1 (58.8 to 69.6)	589	68.9 (63.5 to 74.7)
10y to 15y	563.70	410	72.7 (65.9 to 80.1)	343	60.8 (54.6 to 67.6)
All children	1952.62	4208	215.5 (209.1 to 222.0)	1504	77.0 (73.2 to 81.0)

Figure 2. Acute febrile illness and dengue fever during the study period, by month

Acute dengue fever

A total of 74/1250 3-day fever episodes were positive for acute dengue fever (Dengue NS1 and/or IgM positive); 68/74 were primary acute dengue fever, and 6/74 were acute secondary dengue fever episodes (Supplemental Table 1). The positivity rate (95% CI) for dengue NS1 and/or IgM in our cohort was 5.9% (4.6% to 7.4%). Spatial autocorrelation analysis suggested a clustering pattern for the dengue fever cases in the study area with a 6% likelihood that the observed clustered pattern could be due to random chance (Moran's Index 0.35, z-score 1.8, p=0.06, Fig 3). We observed higher number of acute dengue fever episodes during August to December (monsoon and post-monsoon seasons) with the highest risk in the month of October. Number of dengue fevers were higher in 2018 compared to 2019 (Fig 2).

Figure 3. Geographic distribution of acute dengue fever cases in the study area

Figure legend: Moran's Index = 0.353, Variance = 0.04, z-score= 1.85, p-value = 0.06

Given the z-score of 1.85, there is a less than 10% likelihood that this clustered pattern could be the result of random chance.

Overall incidence (95%CI) of acute dengue fever was 37.9 (29.8 to 47.6) per 1000 PY, primary dengue fever was 34.8 (27.0 to 44.2) per 1000 PY and secondary dengue fever was 3.1 (1.1 to 6.7) per 1000 PY. Incidence of acute dengue fever was observed to be highest among children aged 5 to 10 years (50.4, 95% CI 36.5 to 67.8 per 1000 PY, Table 3). The mean duration of acute dengue fever episode was 6.9 (1.7) days. Only one child had two episodes of acute dengue fever at ages of 5 year 3 months and 6 year 5 months. Two children with acute dengue fever episodes were hospitalized, and mean duration of hospitalization was 8 days. All children with dengue fever recovered; no dengue fever associated deaths were observed (Supplemental Table 2).

Table 3. Incidence of dengue fever by age-categories in the cohort (n = 984)

Age group in years	Person years	Acute dengue fever		Acute dengue fever – primary		Acute dengue fever – secondary	
		No. of episode	Incidence (95%CI) per 1000 PY	No. of episode	Incidence (95%CI) per 1000 PY	No. of episode	Incidence (95%CI) per 1000 PY
6m to <5y	535.08	20	37.38 (22.83 to 57.72)	19	35.51 (21.38 to 55.45)	1	1.87 (0.05 to 10.41)
5y to <10y	853.84	43	50.36 (36.45 to 67.83)	40	46.85 (33.47 to 63.79)	3	3.51 (0.73 to 10.27)
10y to 15y	563.70	11	19.51 (9.74 to 34.91)	9	15.97 (7.30 to 30.31)	2	3.55 (0.43 to 12.82)
All children	1952.62	74	37.90 (29.76 to 47.57)	68	34.83 (27.04 to 44.15)	6	3.07 (1.13 to 6.69)

Of all the acute dengue fever episodes, in 24 cases OD value of dengue NS1 was found to be >1.8 and were subjected to molecular typing of dengue virus (DENV). Dengue PCR was positive in 16 of the 24 specimens (66.7%). The median viral load of dengue in the specimens measured by PCR was estimated to be 138 (IQR 9.25 to 505) x 10^4 copies/ml. In the tested

specimens, the predominant serotype identified was DEN3 in 62.5% (15/24), followed by DEN1 in 4.2% (1/24). We did not identify any DEN2 or DEN4 serotype (Figure 1).

Beyond dengue, in the 1250 AFI episodes, 18 were found to be positive for Salmonella Typhi, 7 were found to be positive for Salmonella Paratyphi and 7 were found to be positive for Chikungunya.

Discussion

Our pediatric cohort showed high burden of acute dengue fever in low-middle income urban settings in Delhi. The incidence of dengue fever was highest among children aged 5 to 10 years. All children recovered without any associated major complications or death. Acute dengue fever episodes were higher in the months of August to December; more dengue fever episodes were recorded in 2018 against 2019. We observed a clustering pattern in the dengue fever cases in the community. In our study sample, DEN3 was the most encountered strain.

The estimates of dengue fever in children from our study seems to be similar or higher compared with community-based cohorts from other countries (10-13). In a prospective cohort of 3000 Latin American children aged 9-16 years followed up during 2010-2011 the reported incidence of dengue fever was 41 (95% CI 32 to 54) per 1000 person-years (11). The incidence of dengue fever in the Nicaraguan pediatric cohort study of 5,545 children aged 2–14 years was 16.1 (95% CI 14.5 to 17.8) per 1,000 person-years (14). In 2012-13, a prospective cohort of subjects ≥6 months old in Cebu City, Philippines reported incidence of symptomatic dengue to be 16.2 per 1000 person-years (12). Between November 2008 and January 2010, in a cohort of 800 children from urban Colombo, Sri Lanka the incidence of dengue fever per 1000 children per year was 33.8 (95% CI 22.4 to 48.8) (15).

But there are limited community-based studies that reported incidence of dengue fever in Indian children. Similar to our effort, the Vellore site of the SEFI cohorts reported the annual incidence rate of dengue in 2017-2018 to be 49.5 per 1000 PY in children aged 6 months to 14 years with fever >3 days (16). Using the serial sero-survey methodology during 2014-2016, a study in a rural area of Pune, Maharashtra estimated the incidence rate of primary dengue infections as 54.2/1000 PY (95% CI 43.0 to 67.3) among children aged 5 to 15 years (17). Despite some heterogeneity due differences in methodology, pooled estimates of our study along with above two studies indicate that the population-based incidence of symptomatic dengue fever might vary from around 35 to 60 per 1000 PY in Indian children under 15 years of age across different geographies (10-13). However, multicentric studies with improved diagnostics and standard methodologies across sites may be helpful to generate more precise country-specific estimates.

For diagnosis of dengue fever, rt-PCR is currently considered as the gold standard although NS1 has high sensitivity and specificity. But even rt-PCR may have its own challenges. It has been seen that rt-PCR may be false negative in cases where dengue virus RNA is detected using transcription-mediated amplification (TMA) technique, and where NS1 is found to be positive. Therefore, it has been suggested that NS1 antigen assay is a valuable method for diagnosis of dengue independent of rt-PCR (18). In limited-resource settings, some researchers even consider NS1 ELISA assay to be superior to rt-PCR due to its high sensitivity, low cost, ease of performance and rapidity (18, 19). NS1 in combination with IgM assay may offer the most sensitive and cost-effective diagnostic modality for dengue diagnosis (19). Previous longitudinal studies in Latin America (11) and in India (16) have used NS1 along with IgM assay to estimate incidence of dengue fever, which is similar to our study. Nonetheless, we acknowledge that rt-PCR in addition to NS1 and IgM could enhance the accuracy of the estimates.

The National Centre for Disease Control reported a higher number of cases in 2018 compared to 2019 in Delhi as was also observed in our study (6). The higher burden of dengue cases as observed during the post-monsoon months of September to December can be attributed to climatic conditions such as mean temperatures and precipitation levels which shortens the extrinsic incubation period of the dengue virus (16, 20). Beyond the seasonal peak, we observed cases of dengue throughout the year suggesting sustained endemic transmission similar to other Southeast Asian countries (21). All four strains of the dengue virus are known to circulate during epidemics in India (22). The community-based study from Vellore, Tamil Nadu, in 2017-18 reported DEN 1 as most common subtype in their population (16). DEN 1 and 2 has been previously reported to be the commonest circulating strains in North India (23). We found DEN 3 as the most common strain in our study sample. The findings corroborate to a recent study in Delhi (24) and may indicate an epidemiological shift. The changing trend of the virus type underscores the importance of continuous disease surveillance.

The study findings have important implications. The high burden of dengue fever specially in the 5 to 10 year old children highlights their vulnerability given the current WHO recommendation to use the available dengue vaccine only in those with a documented past infection or in children above 9 years of age in select areas of high seroprevalence >80% (25). In addition to continued efforts towards development of newer dengue vaccines and evaluating its effectiveness in younger children, it is crucial to develop affordable pre-vaccination screening strategy with high sensitivity and specificity. The significant clustered pattern of cases in our study area may be related to local mosquito breeding patterns in these sites and highlight the importance of promoting vector surveillance and control.

This study, to the best of our knowledge is the first report related to community-based burden of dengue fever in North Indian children. Our study has several limitations. First, our strategy for blood culture precludes capture of dengue fevers with <3-day febrile illness. Second, there were

challenges to conduct blood tests in 20% (254/1250) of the eligible 3-day fever episodes. Based on the 5.9% dengue positivity rate in our cohort, it is possible that around 14/254 of these missed 3-day fever episodes could have tested positive for dengue, giving an overall projected incidence rate of 45/1000 PY. Third, the study was not powered for ascertainment of severity of disease. Fourth, given the median duration of a fever episode of 2 days, a biweekly contact would have allowed better capture of fevers at the cost of operational feasibility. We used a combination of active weekly contacts along with incentivization in form of monthly phone recharge for promoting passive reporting of fever. With our approach, we may have missed some fever episodes as many of these households have a common mobile phone, not always available with the primary caregiver of the child to report fever. Fifth, although rt-PCR is the gold standard for dengue diagnosis, our case ascertainment was based on NS1 and/or IgM positivity. But, given the high sensitivity and specificity (>95%) of the kits compared to rt-PCR, we believe this is not a major source of bias. Lastly, our study population is restricted to children <15 years belonging to low-income urban slum area in Delhi, North India and may not be generalizable to adults or other populations with different characteristics or in other dissimilar geographies.

In conclusion, we think improved methods for disease surveillance along with research for development of newer vaccines particularly for children, together can help to bring down morbidity and mortality related to dengue in our country.

Acknowledgements

We thank the whole team at Clinical and research laboratories at Society for Applied Studies team and the field team for the Tier 1 SEFI study at CHRD SAS for their intensive efforts. We are grateful for the guidance and support provided by Dr. Nita Bhandari, Senior Scientist and Director at Centre for Health Research and Development Society for Applied Studies, New Delhi.

Author Contributions

BS conceived the idea for the study, was involved in data collection, cleaning and analysis, writing the first draft, review and editing and had full access to the study datasets and act as guarantor. MK was involved in data cleaning, statistical analysis, review and editing. AA was involved in data collection and study coordination. AR was involved in geospatial mapping and its interpretation. AC, AD and DM was involved in laboratory analyses and interpretation, review and providing technical inputs to the manuscript. NG and TSC was involved in study supervision, review, editing and providing technical inputs to the manuscript. All authors approved the final version of the paper.

Competing interests

None declared

References

1. Zeng Z, Zhan J, Chen L, Chen H, Cheng S. Global, regional, and national dengue burden from 1990 to 2017: A systematic analysis based on the global burden of disease study 2017. EClinicalMedicine. 2021;32:100712.

2. Stanaway JD, Shepard DS, Undurraga EA, Halasa YA, Coffeng LE, Brady OJ, et al. The global burden of dengue: an analysis from the Global Burden of Disease Study 2013. The Lancet Infectious diseases. 2016;16(6):712-23.

3. Bhatt S, Gething PW, Brady OJ, Messina JP, Farlow AW, Moyes CL, et al. The global distribution and burden of dengue. Nature. 2013;496(7446):504-7.

4. Chakravarti A, Arora R, Luxemburger C. Fifty years of dengue in India. Transactions of the Royal Society of Tropical Medicine and Hygiene. 2012;106(5):273-82.

5. Murhekar M, Joshua V, Kanagasabai K, Shete V, Ravi M, Ramachandran R, et al. Epidemiology of dengue fever in India, based on laboratory surveillance data, 2014-2017. International journal of infectious diseases : IJID : official publication of the International Society for Infectious Diseases. 2019;84s:S10-s4.

6. NVBDCP. Dengue cases and deaths in the country since 2010. In: Programme NVBDC, editor. New Delhi.

7. Shepard DS, Halasa YA, Tyagi BK, Adhish SV, Nandan D, Karthiga KS, et al. Economic and disease burden of dengue illness in India. The American journal of tropical medicine and hygiene. 2014;91(6):1235-42.

8. Ganeshkumar P, Murhekar MV, Poornima V, Saravanakumar V, Sukumaran K, Anandaselvasankar A, et al. Dengue infection in India: A systematic review and meta-analysis. PLoS neglected tropical diseases. 2018;12(7):e0006618.

9. John J, Bavdekar A, Rongsen-Chandola T, Dutta S, Kang G. Estimating the incidence of enteric fever in children in India: a multi-site, active fever surveillance of pediatric cohorts. BMC public health. 2018;18(1):594.

10. Vong S, Khieu V, Glass O, Ly S, Duong V, Huy R, et al. Dengue incidence in urban and rural Cambodia: results from population-based active fever surveillance, 2006-2008. PLoS neglected tropical diseases. 2010;4(11):e903.

11. Dayan G, Arredondo JL, Carrasquilla G, Deseda CC, Dietze R, Luz K, et al. Prospective cohort study with active surveillance for fever in four dengue endemic countries in Latin America. The American journal of tropical medicine and hygiene. 2015;93(1):18-23.

12. Alera MT, Srikiatkhachorn A, Velasco JM, Tac-An IA, Lago CB, Clapham HE, et al. Incidence of Dengue Virus Infection in Adults and Children in a Prospective Longitudinal Cohort in the Philippines. PLoS neglected tropical diseases. 2016;10(2):e0004337.

13. Gordon A, Kuan G, Mercado JC, Gresh L, Avilés W, Balmaseda A, et al. The Nicaraguan pediatric dengue cohort study: incidence of inapparent and symptomatic dengue virus infections, 2004-2010. PLoS neglected tropical diseases. 2013;7(9):e2462.

14. Gordon A, Kuan G, Mercado JC, Gresh L, Avilés W, Balmaseda A, et al. The Nicaraguan Pediatric Dengue Cohort Study: Incidence of Inapparent and Symptomatic Dengue Virus Infections, 2004–2010. PLOS Neglected Tropical Diseases. 2013;7(9):e2462.

15. Tissera H, Amarasinghe A, De Silva AD, Kariyawasam P, Corbett KS, Katzelnick L, et al. Burden of dengue infection and disease in a pediatric cohort in urban Sri Lanka. Am J Trop Med Hyg. 2014;91(1):132-7.

16. Rose W, Sindhu KN, Abraham AM, Kang G, John J. Incidence of dengue illness among children in an urban setting in South India: A population based study. International journal of infectious diseases : IJID : official publication of the International Society for Infectious Diseases. 2019;84s:S15-s8.

17. Shah PS, Alagarasu K, Karad S, Deoshatwar A, Jadhav SM, Raut T, et al. Seroprevalence and incidence of primary dengue infections among children in a rural region of Maharashtra, Western India. BMC Infectious Diseases. 2019;19(1):296.

18. Arya SC, Agarwal N. NS1 detection in addition to reverse transcriptase PCR and transcription-mediated amplification of dengue virus RNA in acutely ill patients. Journal of clinical microbiology. 2009;47(6):1983; author reply

19. Anand AM, Sistla S, Dhodapkar R, Hamide A, Biswal N, Srinivasan B. Evaluation of NS1 Antigen Detection for Early Diagnosis of Dengue in a Tertiary Hospital in Southern India. Journal of clinical and diagnostic research : JCDR. 2016;10(4):Dc01-4.

20. Mutheneni SR, Morse AP, Caminade C, Upadhyayula SM. Dengue burden in India: recent trends and importance of climatic parameters. Emerg Microbes Infect. 2017;6(8):e70.

21. Morales I, Salje H, Saha S, Gurley ES. Seasonal Distribution and Climatic Correlates of Dengue Disease in Dhaka, Bangladesh. Am J Trop Med Hyg. 2016;94(6):1359-61.

22. Gupta N, Srivastava S, Jain A, Chaturvedi UC. Dengue in India. Indian J Med Res. 2012;136(3):373-90.

23. Savargaonkar D, Sinha S, Srivastava B, Nagpal BN, Sinha A, Shamim A, et al. An epidemiological study of dengue and its coinfections in Delhi. International journal of infectious diseases : IJID : official publication of the International Society for Infectious Diseases. 2018;74:41-6.

24. Parveen N, Islam A, Tazeen A, Hisamuddin M, Abdullah M, Naqvi IH, et al. Circulation of single serotype of Dengue Virus (DENV-3) in New Delhi, India during 2016: A change in the epidemiological trend. Journal of infection and public health. 2019;12(1):49-56.

25. Organization WH. Weekly epidemiological record - Dengue vaccines: WHO position paper. 7 SEPTEMBER 2018, 93th YEAR.

Figure 1

Click here to access/download;Figure;Figure1. Study flow.tif

Figure 1. Study Flowchart

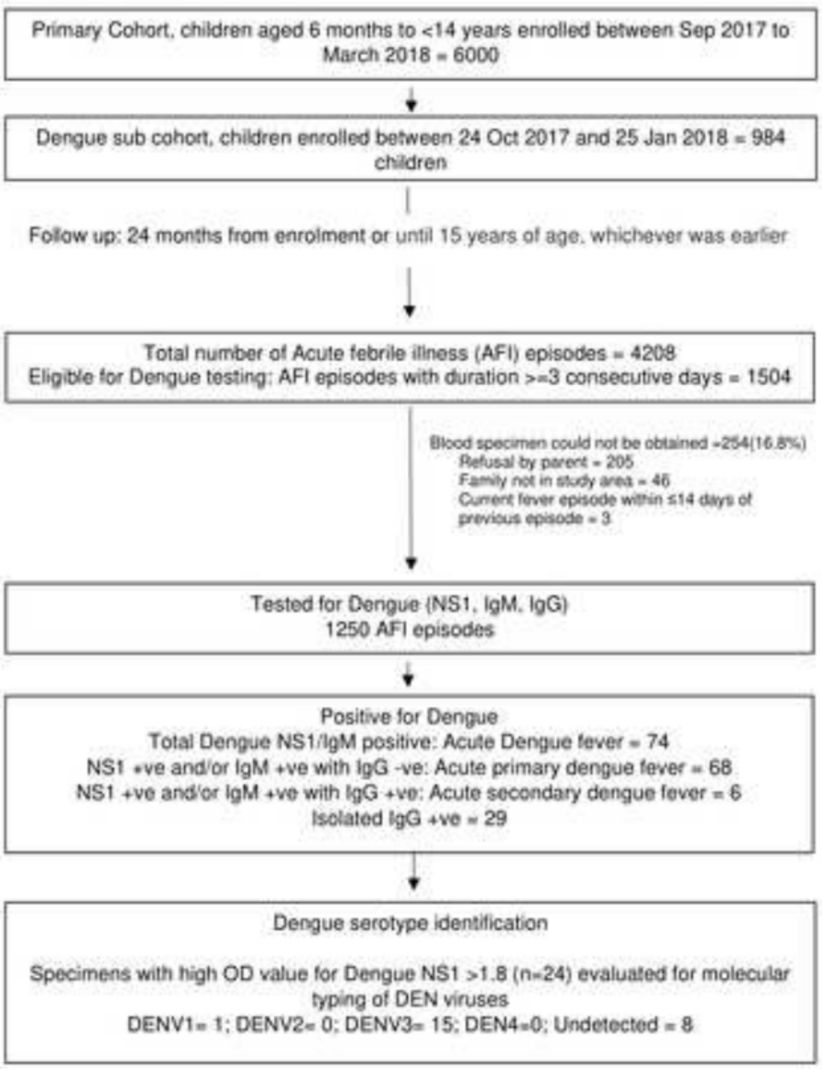

Figure 2

Click here to access/download;Figure;Figure 2. Dengue trends.tif

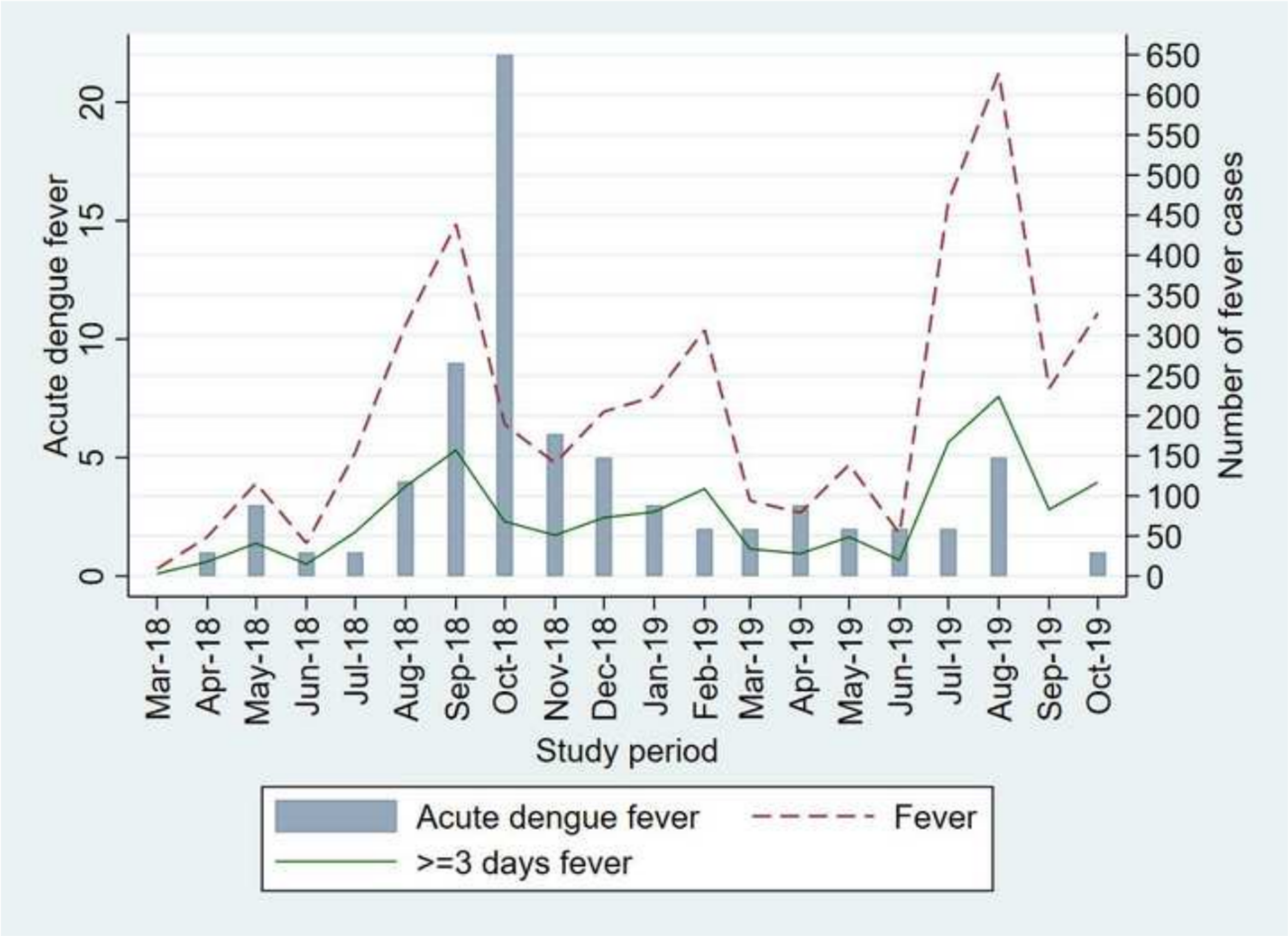

Figure 3

Click here to access/download;Figure;Fig 3.tif

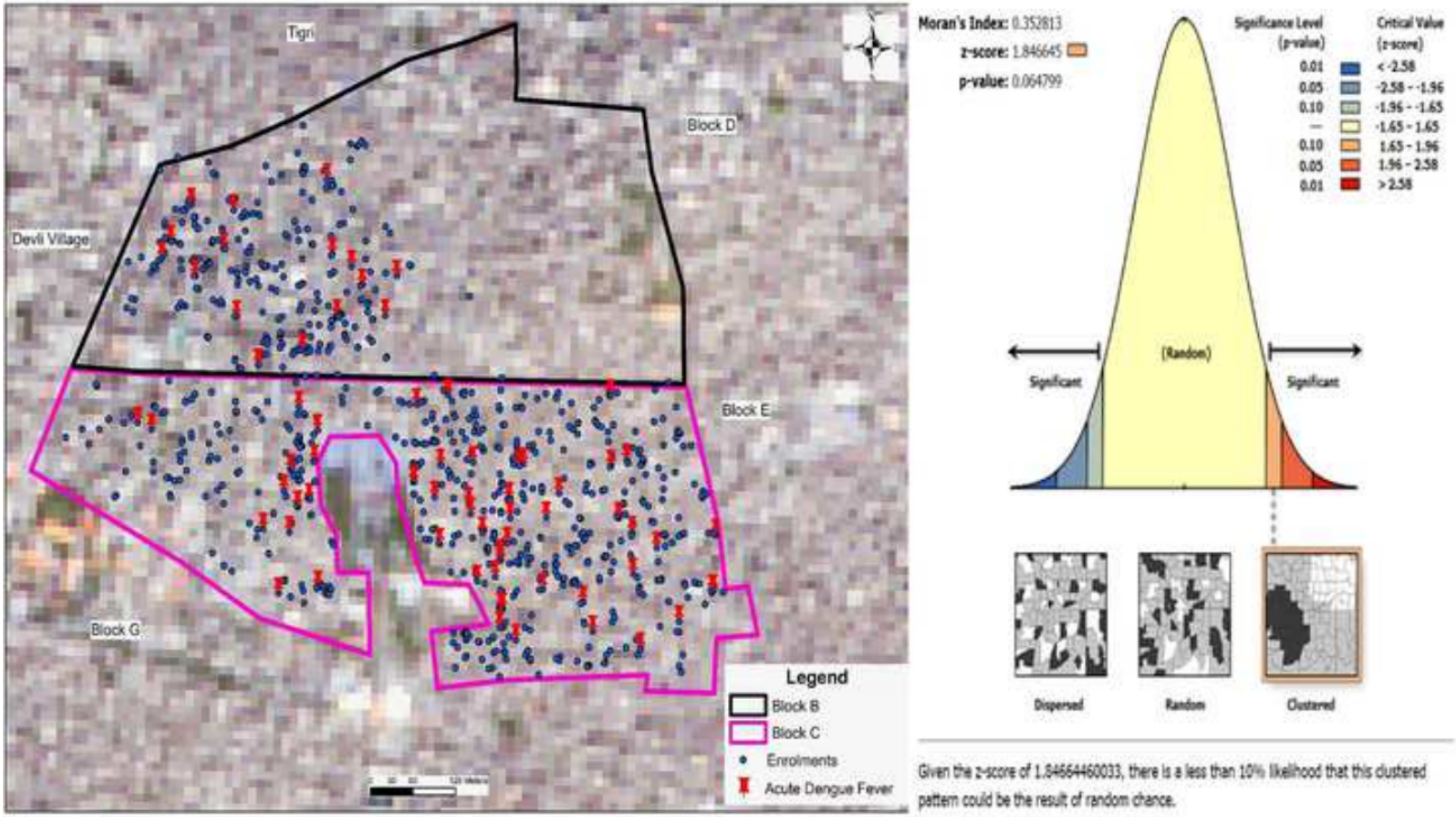

Click here to access/download
Supporting Information
STROBE_checklist_cohort.docx

Supporting Information

Click here to access/download
Supporting Information
Dengue_Sefi_Appendix_07Feb.docx

AUTHOR RESPONSE LETTER

Dear Editor
PLOS Neglected Tropical Diseases

We thank you for your response and invitation to resubmit the paper in the PLOS Neglected Tropical Diseases.
We have taken note of the comments and suggestions made by you and the reviewers and have revised the manuscript accordingly.
As per the editorial requirements, our responses to the comments are presented using a tabular structure with the original editorial / reviewer's comments and authors responses. Where revision to the manuscript text has been made, the text is copied into this letter with yellow highlight along with *page numbers and section in italics* (as they appear in the new/clean version).
Both this clean version of the revised manuscript as well as a version with the changes tracked are also submitted.

Best regards,
Temsunaro Rongsen Chandola

Reviewers' comments	Response
Key Review Criteria Required for Acceptance<br><br>**METHODS**	
**Reviewer #1**	
1) The authors need to describe more on the analysis, especially the stats analysis.	We have elaborated on statistical analysis in *Methods, Statistical analysis section, page 7-8*.
2) What are the diagnosis of secondary and primary infection, why are they probable?	We agree with your comment and have revised to omit the term 'probable'.<br>We have mentioned in the *methods section page 8* that "In addition to NS1 and/or IgM, fever episodes that were positive for dengue IgG were identified as acute secondary dengue fever and those negative for IgG were acute primary dengue fever."<br><br>In our study the median day of blood specimen collection was day 3 of fever. This definition of primary and secondary dengue fever is in line with previously published manuscripts.<br>Ref: Changal KH, Raina AH, Raina A, et al. Differentiating secondary from primary dengue using IgG to IgM ratio in early dengue: an observational hospital based clinico-serological study from North India. *BMC Infect Dis*. 2016;16(1):715. Published 2016 Nov 28. doi:10.1186/s12879-016-2053-6
3) I am curious about the severity of the disease. Why do the author not classify the severity?	Our community-based study was not powered to comment on severity of dengue fever, and this was not the primary objective of the study. Our study was planned with the primary objective to estimate the incidence of lab-confirmed acute dengue fever using ELISA based assays in children under 15 years of age nested within the Surveillance of Enteric Fever in India (SEFI) cohort.

	Regarding severity of illness, we have mentioned in *results section page 12* that "Two children with acute dengue fever episodes were hospitalized, and mean duration of hospitalization was 8 days. All children with dengue fever recovered; no dengue fever associated deaths were observed."<br>Confirmation of severity of dengue (DF, DHF 1, 2 and 3) would require additional blood investigations in all, which was beyond the scope of the study.<br>However, we agree that this information would be interesting. We have mentioned this as a limitation in the Discussion section.
**Reviewer #2**	
The diagnosis of dengue in this study depends on commercial NS1 testing and IgM testing, which are inadequate within the first 3 days of fever to capture all cases. Ideally all samples should have undergone RT-PCR in addition to NS1 testing. Therefore, the data presented here may still be an underestimate.	Thank you for your comment.<br><br>We agree that in our study the diagnosis of dengue is based on NS1 and IgM assays. Therefore, we have reworded our primary objective in *Introduction section page 5* as "Our study was planned with the primary objective to estimate the incidence of lab-confirmed acute dengue fever using ELISA based assays in children under 15 years of age." We agree that ideally, rt-PCR in addition to NS1 and IgM testing would be valuable as rt-PCR is currently the gold standard for diagnosis. We have therefore mentioned that our estimate may be an underestimate this in the limitation section. However, in endemic settings such as ours, given the high sensitivity (98.3%) and specificity (100%) of the Dengue NS1 Microlisa kits compared to rt-PCR for Dengue diagnosis (as mentioned in the J. Mitra kit inserts used in the study) we believe that the issue of underestimation is not a major problem in our study.<br><br>In a landmark prospective cohort study to estimate incidence of dengue fever in four Latin American countries (20 sites), Dayan G et al. 2015 have used similar approach as our study. In this study cases with a positive NS1 ELISA antigen were classified as "virologically confirmed dengue". Suspected dengue cases with a positive IgM ELISA result were classified as probable dengue.<br><br>Ref: Dayan G, Arredondo JL, Carrasquilla G, Deseda CC, Dietze R, Luz K, Costa MSN, Cunha RV, Rey LC, Morales J, Reynales H, Miranda M, Zambrano B, Rivas E, Garbes P, Noriega F. Prospective cohort study with active surveillance for fever in four dengue endemic countries in Latin America. Am J Trop Med Hyg. 2015 Jul;93(1):18-23.<br><br>Another recent study has also used similar methodology as ours.<br><br>Ref: Rose W, Sindhu KN, Abraham AM, Kang G, John J. Incidence of dengue illness among children in an urban setting in South India: A population based study. Int J Infect Dis. 2019;84s:S15-s8.

	Moreover, studies have even reported issues with rt-PCR as well, for dengue diagnosis. There are reports showing rt-PCR to be false negative in cases where dengue virus (DENV) RNA is detected using transcription-mediated amplification (TMA) technique where NS1 is also found to be positive. Therefore, NS1 is a robust assay, and it may have value beyond rt-PCR assay in Dengue diagnosis.<br>Ref: Arya SC, Agarwal N. NS1 detection in addition to reverse transcriptase PCR and transcription-mediated amplification of dengue virus RNA in acutely ill patients. J Clin Microbiol. 2009;47(6):1983. doi:10.1128/JCM.00719-09.<br><br>Anand MA et al. 2016 have reported that "Both NS1 and RT-PCR are useful for early dengue diagnosis, although in terms of cost, ease of performance and rapidity, NS1 is superior to RT-PCR. NS1 in combination with IgM assay offers the most sensitive and cost-effective diagnostic modality for dengue."<br>Ref: Anand AM, Sistla S, Dhodapkar R, Hamide A, Biswal N, Srinivasan B. Evaluation of NS1 Antigen Detection for Early Diagnosis of Dengue in a Tertiary Hospital in Southern India. J Clin Diagn Res. 2016;10(4):DC01-DC4.<br><br>Based on the above arguments, we think our study methodology is well justified. To sum up we have added in the *DISCUSSION SECTION page 14* that "For diagnosis of dengue fever, rt-PCR is currently considered as the gold standard although NS1 has high sensitivity and specificity. But even rt-PCR may have its own challenges. It has been seen that rt-PCR may be false negative in cases where dengue virus RNA is detected using transcription-mediated amplification (TMA) technique, and where NS1 is found to be positive. Therefore, it has been suggested that NS1 antigen assay is a valuable method for diagnosis of dengue independent of rt-PCR (18). In limited-resource settings, some researchers even consider NS1 ELISA assay to be superior to rt-PCR due to its high sensitivity, low cost, ease of performance and rapidity (18, 19). NS1 in combination with IgM assay may offer the most sensitive and cost-effective diagnostic modality for dengue diagnosis (19). Previous longitudinal studies in Latin America (11) and in India (16) have used NS1 along with IgM assay to estimate incidence of dengue fever, which is similar to our study. Nonetheless, we acknowledge that rt-PCR in addition to NS1 and IgM could enhance the accuracy of the estimates."
Selecting a subset of NS1 positive cases for RT-PCR (again this sample is very small) is rather non-sensical as NS1 negative patients can be positive with RT-PCR. It seems authors have done the RT-PCR not as a diagnostic test for dengue, but to determine the infecting serotype. Again, if this is the case a sample size calculation is needed to confidently say that DENV-3 predominates the current epidemic and this small sample is	We agree with you that in our study RT-PCR was used not as a diagnostic test for dengue, but to determine the infecting serotype in a subset of NS1 positive specimens.<br><br>We agree that it is possible that in a small proportion of NS1 negative participants can be RT-PCR positive. But, NS1 is already a well-accepted assay for dengue diagnosis with high sensitivity and specificity, and many validation studies have been done before. Therefore, we did not see a lot of value addition to do another validity assessment of NS1 by comparing with RTPCR

representative of all dengue cases in "North India" to come to this conclusion.	assay in a subset of all enrolled children (irrespective of NS1 positivity) in our study population.<br><br>We have mentioned at the end of the *introduction section page 5* that "As an exploratory objective we reported the most common circulating serotype in the population in a subsample." Given that it is an exploratory or secondary objective, it does not always necessitate sample size calculation. Our sample size calculation was done for the primary objective i.e., to estimate incidence of dengue. For serotype identification, we conducted molecular testing in all specimens which had a NS1 OD value of ≥1.8. This was based on the experience at Department of Virology, AIIMS, New Delhi, that it is difficult to get a positive signal for a specific dengue serotype in multiplex PCR assays in specimens with lower NS1 OD values. We have added in the *Methods section page 7* that "This was based on prior experience that it is difficult to get a positive signal for a specific dengue serotype in multiplex PCR assays in specimens with lower NS1 OD values."<br><br>We agree that as the serotype detection was done is a sub-sample, we are not powered enough to make conclusive statements. We have now changed it to "In our study sample, DEN3 was the most encountered strain."<br><br>We have limited our generalizability statements to low-middle income populations in Delhi.
On top of the above limitations, the study only samples a limited geographical region in South Delhi predominantly from low socioeconomic background. Therefore, this data is not representative of "North India" or even greater Delhi.	We agree. We have limited our generalizability statements to low-middle income populations in Delhi
**Reviewer #3**	
The authors have studied the incidence of Dengue fever in cohort of children in low income urban community in Delhi. This was a sub-cohort of the study on surveillance of enteric fever in India done at the same site. The sample size was adequate for the estimating the incidence which was the main objective. The age group studied does not include children above 15 years.<br>Most of the infections were primary infections. The sample recruited was not adequate to study the incidence of severe forms of dengue fever.	Thank you for your positive comments.<br><br>We agree with you that sample recruited was not adequate to study the incidence of severe forms of dengue fever. We have mentioned this as a limitation in the Discussion section.
**RESULTS**	
**Reviewer #1**	
1) There is no information of missing data	Thank you for your comments. We have updated the information on missing data in *Figure 1*.

2) How many did participants withdraw from the study or miss the follow ups due to moving out the area?	Of the 1504 fever episodes eligible for Dengue testing we could not obtain blood in 254 (16.8%). The reasons were Refusal by parent in 205 cases, family not in study area in 46 cases and current fever episode within ≤14 days of previous episode in 3 cases.
**Reviewer #2**	
The above limitations highlighted in methods makes the estimates in results unreliable	We have addressed this concern above.<br>We believe our study provides realistic and valid estimates of lab-confirmed dengue fever among children in the population.
**Reviewer #3**	
It is not clear if other causes of fever were excluded. The clinical details of any accompanying symptoms is not mentioned. This is important as a significant proportion were prescribed antibiotics.	Thanks for your comments.<br><br>The primary objective of our community-based study was to estimate the incidence of lab-confirmed acute dengue fever using ELISA based assays in children under 15 years of age nested within the Surveillance of Enteric Fever in India (SEFI) cohort. However, we looked at data of the 1250 AFI episodes tested for dengue. We have added in *Results section page 13* that "Beyond dengue, in the 1250 AFI episodes, 18 were found to be positive for Salmonella Typhi, 7 were found to be positive for Salmonella Paratyphi and 7 were found to be positive for Chikungunya."<br><br>In the cases with dengue fever, other clinical symptoms were seldom reported. However, we have added this information in a tabular format for your reference in ***Supplemental Table 2***. This is also cited in the Results section of the manuscript.
Cases which had only IgM positivity with negative NS1 antigen could have had dengue fever in recent past as IgM can stay in circulations for a couple of months. It will be better to give the number of cases which were in this category.	We agree with you. We have added a Table in ***Supplemental Table 1*** titled "Distribution of different dengue assay results and its classification" with the information as suggested. This is also cited in the Results section of the manuscript.
**CONCLUSIONS**	
**Reviewer #1**	
(No Response)	–
**Reviewer #2**	
The limitations are not properly acknowledged. With this data the authors cannot fulfil the aims of their study since the overall dengue incidence is likely to be underestimated without RT-PCR for all samples.	Thank you for your comment.<br>We have elaborated on your comments in earlier sections.<br>In India, community-based estimates of incidence of Dengue fever are very limited. Although we acknowledge that RT-PCR is the gold standard for diagnosis of dengue, our estimates based on NS1 and IgM ELISA assays are realistic and valid for among children in the population. Our argument is supported by previous literature and similar publications in the field.
**Reviewer #3**	
Yes. Limitations have been discussed. Conclusion stated in the last paragraph are	Thank you for your positive comments.<br>We agree with you and the *conclusion page 16* has been modified accordingly as "In conclusion, we think improved methods for

not a part of this study since vector surveillance has not been done.	disease surveillance along with research for development of newer vaccines particularly for children, together can help to bring down morbidity and mortality related to dengue in our country."
**EDITORIAL AND DATA PRESENTATION MODIFICATIONS?**	
**Reviewer #1**	
Minor comments<br>1) Please consistently use small letter for dengue such as "several new Dengue vaccines"	Thank you for your comment.<br>We have addressed the suggestion in the entire body of manuscript.
**Reviewer #2**	
(No Response)	_
**Reviewer #3**	
Minor revision	Thank you for your comment.<br>We have worked on the language and consistency across the body of the manuscript.
**SUMMARY AND GENERAL COMMENTS**	
**Reviewer #1**	
(No Response)	_
**Reviewer #2**	
Overall, this article has limited value to an international audience as it only focusses on low socioeconomic groups in a limited geographical region in South Delhi. To say this is representative of dengue incidence in "Northern India" (as mentioned in the title), is misleading. This data may not even be representative of greater metropolitan Delhi (since not all socioeconomic groups are represented) and on top of that may underestimate the true incidence of dengue due to issues in diagnosis highlighted above.	Thank you for your views. But we think otherwise.<br>In India majority of the population (~80%) belong to low-middle socioeconomic group. Moreover, population-based cohorts are the method of choice to derive incidence estimates but not always feasible to conduct at a time in all parts of a country such as India. The population characteristics of our study area in South Delhi in terms of socioeconomic status, literacy and religion is representative of around 70-80% of similar populations in India. However, we understand geographical conditions are different in other regions and can influence dengue disease rates. Although we acknowledge that RT-PCR is the gold standard for diagnosis of dengue, our estimates based on NS1 and IgM ELISA assays are realistic and valid for among children in the population (based on prior evidence). **Therefore, we believe, our study shows valid evidence of high burden of acute dengue fever in low-middle income urban populations in Delhi and adds value to available evidence.**<br>We have also compared our study with previous Indian studies in the *Discussion section page 14* and have mentioned (based on available data) that "Despite some heterogeneity due differences in methodology, pooled estimates of our study along with above two studies indicate that the population-based incidence of symptomatic dengue fever might vary from around 35 to 60 per 1000 PY in Indian children under 15 years of age across different geographies (10-13). However, multicentric studies with improved diagnostics and standard methodologies across sites may be helpful to generate more precise country-specific estimates."
**Reviewer #3**	

It is a well written article with public health importance. It emphasizes the burden of dengue fever in low income community of North India. The authors report change in circulating strain to DEN 3 which highlights the need for continued surveillance.	We thank you for your positive comments and agree with you on the need for continued surveillance.

Click here to access/download
Revised Article with Changes Highlighted
Manuscript_14 Feb_TRACK.docx

PNTD-D-21-01656_R1_reviewer.pdf

ORIGINALITY REPORT

37%
SIMILARITY INDEX

MATCH ALL SOURCES (ONLY SELECTED SOURCE PRINTED)

★journals.plos.org
Internet

9%

EXCLUDE QUOTES	OFF	EXCLUDE MATCHES	OFF
EXCLUDE BIBLIOGRAPHY	OFF		
