# Supplementary material for: Incidence of lab-confirmed dengue fever in a pediatric cohort in Delhi, India
Source: PLoS Negl Trop Dis. 2022 Apr 7;16(4):e0010333. doi: 10.1371/journal.pntd.0010333 (PMC9017938; doi:10.1371/journal.pntd.0010333)
Supplement: Attachment — Submitted filename: Responsetoreviewers letter_07Feb.docx [file pntd.0010333.s004.docx]

**AUTHOR RESPONSE LETTER**

Dear Editor

PLOS Neglected Tropical Diseases

We thank you for your response and invitation to resubmit the paper in the PLOS Neglected Tropical Diseases.

We have taken note of the comments and suggestions made by you and the reviewers and have revised the manuscript accordingly.

As per the editorial requirements, our responses to the comments are presented using a tabular structure with the original editorial / reviewer’s comments and authors responses. Where revision to the manuscript text has been made, the text is copied into this letter with yellow highlight along with *page numbers and section* *in italics* (as they appear in the new/clean version).

Both this clean version of the revised manuscript as well as a version with the changes tracked are also submitted.

Best regards,

Temsunaro Rongsen Chandola

| **Reviewers’ comments** | **Response** |
| --- | --- |
| Key Review Criteria Required for Acceptance  **METHODS** | |
| **Reviewer #1** |  |
| 1) The authors need to describe more on the analysis, especially the stats analysis. | We have elaborated on statistical analysis in *Methods, Statistical analysis section, page 7-8*. |
| 2) What are the diagnosis of secondary and primary infection, why are they probable? | We agree with your comment and have revised to omit the term ‘probable’.  We have mentioned in the *methods section page 8* that “In addition to NS1 and/or IgM, fever episodes that were positive for dengue IgG were identified as acute secondary dengue fever and those negative for IgG were acute primary dengue fever.”  In our study the median day of blood specimen collection was day 3 of fever. This definition of primary and secondary dengue fever is in line with previously published manuscripts.  Ref: [Changal KH, Raina AH, Raina A, et al. Differentiating secondary from primary dengue using IgG to IgM ratio in early dengue: an observational hospital based clinico-serological study from North India. *BMC Infect Dis*. 2016;16(1):715. Published 2016 Nov 28. doi:10.1186/s12879-016-2053-6](https://bmcinfectdis.biomedcentral.com/track/pdf/10.1186/s12879-016-2053-6.pdf) |
| 3) I am curious about the severity of the disease. Why do the author not classify the severity? | Our community-based study was not powered to comment on severity of dengue fever, and this was not the primary objective of the study. Our study was planned with the primary objective to estimate the incidence of lab-confirmed acute dengue fever using ELISA based assays in children under 15 years of age nested within the Surveillance of Enteric Fever in India (SEFI) cohort.  Regarding severity of illness, we have mentioned in *results section page 12* that “Two children with acute dengue fever episodes were hospitalized, and mean duration of hospitalization was 8 days. All children with dengue fever recovered; no dengue fever associated deaths were observed.”  Confirmation of severity of dengue (DF, DHF 1, 2 and 3) would require additional blood investigations in all, which was beyond the scope of the study.  However, we agree that this information would be interesting. We have mentioned this as a limitation in the Discussion section. |
| **Reviewer #2** |  |
| The diagnosis of dengue in this study depends on commercial NS1 testing and IgM testing, which are inadequate within the first 3 days of fever to capture all cases. Ideally all samples should have undergone RT-PCR in addition to NS1 testing. Therefore, the data presented here may still be an underestimate. | Thank you for your comment.  We agree that in our study the diagnosis of dengue is based on NS1 and IgM assays. Therefore, we have reworded our primary objective in *Introduction section page 5* as “Our study was planned with the primary objective to estimate the incidence of lab-confirmed acute dengue fever using ELISA based assays in children under 15 years of age.” We agree that ideally, rt-PCR in addition to NS1 and IgM testing would be valuable as rt-PCR is currently the gold standard for diagnosis. We have therefore mentioned that our estimate may be an underestimate this in the limitation section. However, in endemic settings such as ours, given the high sensitivity (98.3%) and specificity (100%) of the Dengue NS1 Microlisa kits compared to rt-PCR for Dengue diagnosis (as mentioned in the J. Mitra kit inserts used in the study) we believe that the issue of underestimation is not a major problem in our study.  In a landmark prospective cohort study to estimate incidence of dengue fever in four Latin American countries (20 sites), Dayan G et al. 2015 have used similar approach as our study. In this study cases with a positive NS1 ELISA antigen were classified as “virologically confirmed dengue”. Suspected dengue cases with a positive IgM ELISA result were classified as probable dengue.  Ref: [Dayan G, Arredondo JL, Carrasquilla G, Deseda CC, Dietze R, Luz K, Costa MSN, Cunha RV, Rey LC, Morales J, Reynales H, Miranda M, Zambrano B, Rivas E, Garbes P, Noriega F. Prospective cohort study with active surveillance for fever in four dengue endemic countries in Latin America. Am J Trop Med Hyg. 2015 Jul;93(1):18-23.](https://www.ajtmh.org/view/journals/tpmd/93/1/article-p18.xml?tab_body=pdf)    Another recent study has also used similar methodology as ours.  Ref: [Rose W, Sindhu KN, Abraham AM, Kang G, John J. Incidence of dengue illness among children in an urban setting in South India: A population based study. Int J Infect Dis. 2019;84s:S15-s8.](https://www.ijidonline.com/action/showPdf?pii=S1201-9712%2819%2930044-X)  Moreover, studies have even reported issues with rt-PCR as well, for dengue diagnosis. There are reports showing rt-PCR to be false negative in cases where dengue virus (DENV) RNA is detected using transcription-mediated amplification (TMA) technique where NS1 is also found to be positive. Therefore, NS1 is a robust assay, and it may have value beyond rt-PCR assay in Dengue diagnosis.  Ref: [Arya SC, Agarwal N. NS1 detection in addition to reverse transcriptase PCR and transcription-mediated amplification of dengue virus RNA in acutely ill patients. J Clin Microbiol. 2009;47(6):1983. doi:10.1128/JCM.00719-09.](https://zh.booksc.eu/book/38713217/f69289)  Anand MA et al. 2016 have reported that “Both NS1 and RT-PCR are useful for early dengue diagnosis, although in terms of cost, ease of performance and rapidity, NS1 is superior to RT-PCR. NS1 in combination with IgM assay offers the most sensitive and cost-effective diagnostic modality for dengue.”  Ref: [Anand AM, Sistla S, Dhodapkar R, Hamide A, Biswal N, Srinivasan B. Evaluation of NS1 Antigen Detection for Early Diagnosis of Dengue in a Tertiary Hospital in Southern India. J Clin Diagn Res. 2016;10(4):DC01-DC4.](https://www.ncbi.nlm.nih.gov/pmc/articles/PMC4866096/pdf/jcdr-10-DC01.pdf)  Based on the above arguments, we think our study methodology is well justified. To sum up we have added in the *DISCUSSION SECTION* *page 14* that “For diagnosis of dengue fever, rt-PCR is currently considered as the gold standard although NS1 has high sensitivity and specificity. But even rt-PCR may have its own challenges. It has been seen that rt-PCR may be false negative in cases where dengue virus RNA is detected using transcription-mediated amplification (TMA) technique, and where NS1 is found to be positive. Therefore, it has been suggested that NS1 antigen assay is a valuable method for diagnosis of dengue independent of rt-PCR (18). In limited-resource settings, some researchers even consider NS1 ELISA assay to be superior to rt-PCR due to its high sensitivity, low cost, ease of performance and rapidity (18, 19). NS1 in combination with IgM assay may offer the most sensitive and cost-effective diagnostic modality for dengue diagnosis (19). Previous longitudinal studies in Latin America (11) and in India (16) have used NS1 along with IgM assay to estimate incidence of dengue fever, which is similar to our study. Nonetheless, we acknowledge that rt-PCR in addition to NS1 and IgM could enhance the accuracy of the estimates.” |
| Selecting a subset of NS1 positive cases for RT-PCR (again this sample is very small) is rather non-sensical as NS1 negative patients can be positive with RT-PCR. It seems authors have done the RT-PCR not as a diagnostic test for dengue, but to determine the infecting serotype. Again, if this is the case a sample size calculation is needed to confidently say that DENV-3 predominates the current epidemic and this small sample is representative of all dengue cases in "North India" to come to this conclusion. | We agree with you that in our study RT-PCR was used not as a diagnostic test for dengue, but to determine the infecting serotype in a subset of NS1 positive specimens.  We agree that it is possible that in a small proportion of NS1 negative participants can be RT-PCR positive. But, NS1 is already a well-accepted assay for dengue diagnosis with high sensitivity and specificity, and many validation studies have been done before. Therefore, we did not see a lot of value addition to do another validity assessment of NS1 by comparing with RTPCR assay in a subset of all enrolled children (irrespective of NS1 positivity) in our study population.  We have mentioned at the end of the *introduction section* *page 5* that “As an exploratory objective we reported the most common circulating serotype in the population in a subsample.” Given that it is an exploratory or secondary objective, it does not always necessitate sample size calculation. Our sample size calculation was done for the primary objective i.e., to estimate incidence of dengue. For serotype identification, we conducted molecular testing in all specimens which had a NS1 OD value of ≥1.8. This was based on the experience at Department of Virology, AIIMS, New Delhi, that it is difficult to get a positive signal for a specific dengue serotype in multiplex PCR assays in specimens with lower NS1 OD values. We have added in the *Methods section page 7* that “This was based on prior experience that it is difficult to get a positive signal for a specific dengue serotype in multiplex PCR assays in specimens with lower NS1 OD values.”  We agree that as the serotype detection was done is a sub-sample, we are not powered enough to make conclusive statements. We have now changed it to “In our study sample, DEN3 was the most encountered strain.”  We have limited our generalizability statements to low-middle income populations in Delhi. |
| On top of the above limitations, the study only samples a limited geographical region in South Delhi predominantly from low socioeconomic background. Therefore, this data is not representative of "North India" or even greater Delhi. | We agree. We have limited our generalizability statements to low-middle income populations in Delhi |
| **Reviewer #3** |  |
| The authors have studied the incidence of Dengue fever in cohort of children in low income urban community in Delhi. This was a sub-cohort of the study on surveillance of enteric fever in India done at the same site. The sample size was adequate for the estimating the incidence which was the main objective. The age group studied does not include children above 15 years.  Most of the infections were primary infections. The sample recruited was not adequate to study the incidence of severe forms of dengue fever. | Thank you for your positive comments.  We agree with you that sample recruited was not adequate to study the incidence of severe forms of dengue fever. We have mentioned this as a limitation in the Discussion section. |
| **RESULTS** | |
| **Reviewer #1** |  |
| 1) There is no information of missing data  2) How many did participants withdraw from the study or miss the follow ups due to moving out the area? | Thank you for your comments. We have updated the information on missing data in *Figure 1*.  Of the 1504 fever episodes eligible for Dengue testing we could not obtain blood in 254 (16.8%). The reasons were Refusal by parent in 205 cases, family not in study area in 46 cases and current fever episode within ≤14 days of previous episode in 3 cases. |
| **Reviewer #2** |  |
| The above limitations highlighted in methods makes the estimates in results unreliable | We have addressed this concern above.  We believe our study provides realistic and valid estimates of lab-confirmed dengue fever among children in the population. |
| **Reviewer #3** |  |
| It is not clear if other causes of fever were excluded. The clinical details of any accompanying symptoms is not mentioned. This is important as a significant proportion were prescribed antibiotics. | Thanks for your comments.  The primary objective of our community-based study was to estimate the incidence of lab-confirmed acute dengue fever using ELISA based assays in children under 15 years of age nested within the Surveillance of Enteric Fever in India (SEFI) cohort. However, we looked at data of the 1250 AFI episodes tested for dengue. We have added in *Results section page 13* that “Beyond dengue, in the 1250 AFI episodes, 18 were found to be positive for Salmonella Typhi, 7 were found to be positive for Salmonella Paratyphi and 7 were found to be positive for Chikungunya.”  In the cases with dengue fever, other clinical symptoms were seldom reported. However, we have added this information in a tabular format for your reference in ***Supplemental Table 2***. This is also cited in the Results section of the manuscript. |
| Cases which had only IgM positivity with negative NS1 antigen could have had dengue fever in recent past as IgM can stay in circulations for a couple of months. It will be better to give the number of cases which were in this category. | We agree with you. We have added a Table in ***Supplemental Table 1*** titled “Distribution of different dengue assay results and its classification” with the information as suggested. This is also cited in the Results section of the manuscript. |
| **CONCLUSIONS** | |
| **Reviewer #1** |  |
| (No Response) | _ |
| **Reviewer #2** |  |
| The limitations are not properly acknowledged. With this data the authors cannot fulfil the aims of their study since the overall dengue incidence is likely to be underestimated without RT-PCR for all samples. | Thank you for your comment.  We have elaborated on your comments in earlier sections.  In India, community-based estimates of incidence of Dengue fever are very limited. Although we acknowledge that RT-PCR is the gold standard for diagnosis of dengue, our estimates based on NS1 and IgM ELISA assays are realistic and valid for among children in the population. Our argument is supported by previous literature and similar publications in the field. |
| **Reviewer #3** |  |
| Yes. Limitations have been discussed. Conclusion stated in the last paragraph are not a part of this study since vector surveillance has not been done. | Thank you for your positive comments.  We agree with you and the *conclusion page 16* has been modified accordingly as “In conclusion, we think improved methods for disease surveillance along with research for development of newer vaccines particularly for children, together can help to bring down morbidity and mortality related to dengue in our country.” |
| **EDITORIAL AND DATA PRESENTATION MODIFICATIONS?** | |
| **Reviewer #1** |  |
| Minor comments  1) Please consistently use small letter for dengue such as "several new Dengue vaccines" | Thank you for your comment.  We have addressed the suggestion in the entire body of manuscript. |
| **Reviewer #2** |  |
| (No Response) | _ |
| **Reviewer #3** |  |
| Minor revision | Thank you for your comment.  We have worked on the language and consistency across the body of the manuscript. |
| **SUMMARY AND GENERAL COMMENTS** | |
| **Reviewer #1** |  |
| (No Response) | _ |
| **Reviewer #2** |  |
| Overall, this article has limited value to an international audience as it only focusses on low socioeconomic groups in a limited geographical region in South Delhi. To say this is representative of dengue incidence in "Northern India" (as mentioned in the title), is misleading. This data may not even be representative of greater metropolitan Delhi (since not all socioeconomic groups are represented) and on top of that may underestimate the true incidence of dengue due to issues in diagnosis highlighted above. | Thank you for your views. But we think otherwise.  In India majority of the population (~80%) belong to low-middle socioeconomic group. Moreover, population-based cohorts are the method of choice to derive incidence estimates but not always feasible to conduct at a time in all parts of a country such as India. The population characteristics of our study area in South Delhi in terms of socioeconomic status, literacy and religion is representative of around 70-80% of similar populations in India. However, we understand geographical conditions are different in other regions and can influence dengue disease rates. Although we acknowledge that RT-PCR is the gold standard for diagnosis of dengue, our estimates based on NS1 and IgM ELISA assays are realistic and valid for among children in the population (based on prior evidence). **Therefore, we believe, our study shows valid evidence of high burden of acute dengue fever in low-middle income urban populations in Delhi and adds value to available evidence.**  We have also compared our study with previous Indian studies in the *Discussion section page 14* and have mentioned (based on available data) that “Despite some heterogeneity due differences in methodology, pooled estimates of our study along with above two studies indicate that the population-based incidence of symptomatic dengue fever might vary from around 35 to 60 per 1000 PY in Indian children under 15 years of age across different geographies (10-13). However, multicentric studies with improved diagnostics and standard methodologies across sites may be helpful to generate more precise country-specific estimates.” |
| **Reviewer #3** |  |
| It is a well written article with public health importance. It emphasizes the burden of dengue fever in low income community of North India. The authors report change in circulating strain to DEN 3 which highlights the need for continued surveillance. | We thank you for your positive comments and agree with you on the need for continued surveillance. |
