# Supplementary material for: Incidence of lab-confirmed dengue fever in a pediatric cohort in Delhi, India
Source: PLoS Negl Trop Dis. 2022 Apr 7;16(4):e0010333. doi: 10.1371/journal.pntd.0010333 (PMC9017938; doi:10.1371/journal.pntd.0010333)
Supplement: Attachment — Submitted filename: Responsetoreviewers_09Mar.docx [file pntd.0010333.s006.docx]

**AUTHOR RESPONSE LETTER**

Dear Editor

PLOS Neglected Tropical Diseases

We thank you for your response and invitation to resubmit the paper in the PLOS Neglected Tropical Diseases.

We have taken note of the comments and suggestions made by you and the reviewers and have revised the manuscript accordingly. As per the editorial requirements, our responses to the comments are presented using a tabular structure with the original editorial / reviewer’s comments and authors responses. Where revision to the manuscript text has been made, the text is copied into this letter with yellow highlight along with *page numbers and section in italics* (as they appear in the new/clean version).

Both this clean version of the revised manuscript as well as a version with the changes tracked are also submitted.

Best regards,

Temsunaro Rongsen Chandola

| **Reviewers’ comments** | **Response** |
| --- | --- |
| **Methods** | |
| **Reviewer #1** |  |
| (No Response) | – |
| **Reviewer #2** |  |
| I have reviewed this paper before (Reviewer 2). I do not think the reviewers have adequately addressed my concerns. I accept their rebuttal on diagnostic tests. However many other concerns still remain which are listed below  1. The study title still misleadingly refers to "North India". This samples is not representative of North India. While the authors state that "The population characteristics of our study area in South Delhi in terms of socioeconomic status, literacy and religion is representative of around 70-80% of similar populations in India", there is no reference or evidence to back this claim. | Thank you for your critical and constructive comments.  We agree with you and have limited our generalizability statements to low middle-income populations in Delhi. The title was modified as “Incidence of lab-confirmed dengue fever in a pediatric cohort in Delhi, India”. In the discussion, under Limitations section we have also mentioned that “Our study population is restricted to children <15 years belonging to low-middle income urban area in Delhi, North India and may not be generalizable to adults or other populations with different characteristics or dissimilar geographies.”  The study area Sangam Vihar is one of our field practice areas. We have added in the Methodology section with references that “The study was conducted as a sub-cohort within the Surveillance of Enteric Fever in India (SEFI) cohort among children in Delhi (CTRI/2017/09/009719) (9), in two contiguous blocks of Sangam Vihar, a low-middle income urban and peri-urban neighborhood in the southern district of Delhi (10). In the study area, more than 80% of the population are Hindu by religion, more than half of the women have secondary school education, with an average household monthly income between 10,000 to 20000 INR, similar to the National average (11).”  More details of our study area are published in   1. [Taneja S, Chowdhury R, Dhabhai N, Mazumder S, Upadhyay RP, Sharma S, Dewan R, Mittal P, Chellani H, Bahl R, Bhan MK. Impact of an integrated nutrition, health, water sanitation and hygiene, psychosocial care and support intervention package delivered during the pre-and peri-conception period and/or during pregnancy and early childhood on linear growth of infants in the first two years of life, birth outcomes and nutritional status of mothers: study protocol of a factorial, individually randomized controlled trial in India. Trials. 2020 Dec;21(1):1-8.](https://www.ncbi.nlm.nih.gov/labs/pmc/articles/PMC6995212/pdf/13063_2020_Article_4059.pdf) 2. [Nutritional Interventions to Improve Linear Growth during Infancy in India: Supplementing Lactating Mothers (IMPRINT Trial). CTRI/2018/04/013095 [Registered on: 06/04/2018]](http://www.ctri.nic.in/Clinicaltrials/pdf_generate.php?trialid=24559&EncHid=&modid=&compid=%27,%2724559det%27) |
| 2. The serotyping data lacks adequate power to cme to any conclusion and I disagree with their notion of not needing a sample size calculation | Thank you for your comment.  We agree that as the serotype detection was done is a sub-sample and are not adequately powered.  We have therefore mentioned at the end of the Introduction section that “As an exploratory objective we reported the most common circulating serotype in the population in a subsample.” We have addressed it in *discussion, page 13, line 248* as “In our study sample, DEN3 was the most encountered strain. Our study was not powered to determine the serotype-specific incidence of dengue and therefore should be interpreted with caution.” |
| 3. The cohort was designed for surveillance of enteric fever and some of the data seems to be collected for that purpose with little relevance for dengue. For example in Table 1 the variables mentioned has little relevance to dengue | Thank you for your comments. We agree with you that the cohort was designed for surveillance of enteric fever.  We have modified Table 1 to include only variables relevant to dengue in *Results, characteristics of the study population section, page 9, Table 1 Characteristics of the study population*. |
| 4. The data only refers to a specific geographical area of Delhi and has little relevance for an international audience. | Thank you for your comments. But we think otherwise.  Population based cohort studies are the gold standard for estimating incidence of disease but such studies are very resource intensive and operationally challenging.  This study, to the best of our knowledge is the first report related to community-based burden of dengue fever among children under 15 years of age estimated using a pediatric cohort design in low-middle income settings in Delhi, North India.  The population characteristics of our study area in South Delhi in terms of socioeconomic status, literacy and religion is similar to the National average as per NFHS-4 estimates. We have mentioned in the methods section that “In the study area, more than 80% of the population are Hindu by religion, more than half of the women have secondary school education, with an average household monthly income between 10,000 to 20000 INR, similar to the National average (11).”  We believe that the evidence is valid for urban, peri urban low middle-income populations in Delhi and is applicable to other similar settings in India with contextual relevance. Having said that we also do recognize “Given that India has large variations in geographical and climatic conditions, multicentric studies with standard methodology across sites may help to capture the within country variation in dengue incidence.”  Population-based cohort studies such as ours has important implications both nationally and internationally and can contribute to global burden of disease estimations. |
| **Reviewer #3** |  |
| The main objective of the study was to ascertain the incidence of lab confirmed dengue. The invetigators followedup a cohort of children for episodes of acute febrile illness . The population is clearly described and meticulously followed up  The methods are described in detail. | Thank you for your positive comments |
| **Results** | |
| **Reviewer #1** |  |
| There is a mismatch of children age in different parts of the manuscript such as 5-15, 5-< 14...  There are a lot of self plagiarism as shown in the attached file. | Thank you for comments.  The study enrolled children aged 6 months to less than 14 years and followed them up for either 24 months or until 15 years of age, whichever was earlier. This allowed us to enroll children up to <14 years of age (13 years, 364 days of age) and follow them up for at least a year. We have elaborated it in *Methods, study population section, pages 5 to 6, lines 87 to 91* as “Eligible children aged between 6 months and <14 years who were likely to stay in the study area for two years were enrolled consecutively in the cohort from contiguous households until the sample size was attained. All enrolled children were followed up for 24 months or until 15 years of age, whichever was earlier. The upper age limit at enrollment was restricted to <14 years so that each enrolled child in the cohort be followed up for at least 12 months.”  We have also reviewed the manuscript to be sure on any possible mismatch of children age. |
| **Reviewer #2** |  |
| Please see comments above | Thank you for your comment  We have elaborated on your comments in earlier section. |
| **Reviewer #3** |  |
| The results are presented clearly and figures and images are satisfactory. | Thank you for your positive comments |
| **Conclusions** | |
| **Reviewer #1** |  |
| (No Response) | – |
| **Reviewer #2** |  |
| Please see comments above | Thank you for your comment  We have elaborated on your comments in earlier section. |
| **Reviewer #3** |  |
| The conclusion are supported by the data. The limitations are described . The public health implications are discussed. | Thank you for your positive comments |
| **Editorial and Data Presentation Modifications?** | |
| **Reviewer #1** |  |
| (No Response) | – |
| **Reviewer #2** |  |
| (No Response) | – |
| **Reviewer #3** |  |
| The authors have revised the document as per modifications suggested. | Thank you for your comments |
| **Summary and General Comments** | |
| **Reviewer #1** |  |
| (No Response) | – |
| **Reviewer #2** |  |
| (No Response) | – |
| **Reviewer #3** |  |
| Well designed study | Thank you for your positive comments |
